# Article 3: 1-year impact of supervision, performance assessment, and recognition strategy (SPARS) on prescribing and dispensing quality in Ugandan health facilities

**DOI:** 10.1186/s40545-020-00248-w

**Published:** 2020-09-01

**Authors:** Birna Trap, Moses N. Sembatya, Monica Imi, Morries Seru, Anita K. Wagner, Dennis Ross-Degnan

**Affiliations:** 1 Medicines, Technologies and Pharmaceuticals Services (MTaPS) program, Katmandu, Nepal; 2 Management Sciences for Health, Plot 15, Princess Anne Drive, Bugolobi, P.O. Box 71419, Kampala, Uganda; 3 Pharmacy Division, Ministry of Health, Lourdel Road, Wandegeya, Kampala, Uganda; 4 Harvard Pilgrim Health Care Institute, 133 Brookline Avenue 6th Floor, Boston, MA 02215 USA

**Keywords:** SPARS, Supportive supervision, Prescribing quality, Dispensing quality, Medicine use, Standard treatment guidelines, Appropriate medicine use indicators, Uganda

## Abstract

**Background:**

To strengthen appropriate medicine use (AMU) including the prescribing and dispensing quality at public sector health facilities in Uganda, the Ministry of Health introduced a multipronged approach known as the Supervision, Performance Assessment, and Recognition Strategy (SPARS). This paper assesses the impact of the first year of SPARS implementation on key AMU indicators.

**Methods:**

District-based health workers trained as supervisors provide in-service training in medicines management complemented by indicator-based performance assessment and targeted supervision during each SPARS facility visit. From 2010 to 2013, health facilities that started the SPARS intervention were assessed during the first and last visit during a period of 12 months of implementing SPARS. This study examines 12 AMU indicators with 57 individual outcomes covering prescribing and dispensing quality. We also explored factors influencing 1-year improvement.

**Results:**

We found an overall increase in AMU indicators of 17 percentage points (*p* < 0.000) between the first and last visit during a period of 12 months of supervisions, which was significant in all levels of health care facilities and in both government and private not-for-profit faith-based sectors. Appropriate dispensing (25 percentage points, *p* < 0.005) improved more than appropriate prescribing (12 percentage points, *p* = 0.13). Specific facilities that reached an average score of over 75% across all AMU measures within the first year of supervision improved from 3 to 41% from the first visit (baseline). The greatest overall impact on AMU occurred in lower-level facilities; the level of improvement varied widely across indicators, with the greatest improvements seen for the lowest baseline measures. Supervision frequency had a significant impact on level of improvement in the first year, and private not-for-profit faith-based health facilities had notably higher increases in several dispensing and prescribing indicator scores than public sector facilities.

**Conclusions:**

The multipronged SPARS approach was effective in building appropriate medicine use capacity, with statistically significant improvements in AMU overall and almost all prescribing and dispensing quality measures after 12 months of supervision. We recommend broad dissemination of the SPARS approach as an effective strategy to strengthen appropriate medicine use in low-income countries.

## Background

The Lancet Commission on Essential Medicines Policies identified appropriate medicine use (AMU) as one of five critically important pharmaceutical policy areas for achieving universal health coverage [1]. Inappropriate medicine use can undermine the potential benefits of universal coverage by reducing treatment effectiveness, increasing antimicrobial resistance, jeopardizing health system financing, and risking harm to patients. The World Health Organization (WHO) estimates that more than half of all medicines are prescribed, dispensed, or sold inappropriately [2, 3]. A systematic review of 43 studies from eleven African countries found significant deviation in prescribing indicators from WHO reference targets [2]; for example, the average number of medicines prescribed has increased over the last decade from 2.4 to 3.5 medicines per patient, with increased use of antibiotics and treatment according to standard treatment guidelines (STGs) for only 30 to 40% of patients in low- to middle-income countries [2–4].

The need to strengthen AMU in Uganda has been well documented. A national survey from 2010 found an average of 3.2 medicines was prescribed per patient, 68% of all patients received one or more antibiotics during an encounter, only a third of patients with diarrhea were treated in accordance with STGs, and only 28% of medicines dispensed were appropriately labeled [5]. These problems occurred despite several decades of policies encouraging use of national STGs, the creation of medicines therapeutic committees in all hospitals, well-established training curricula and multiple health worker training workshops.

Barriers to AMU are many and complex, involving problems that occur at facility, staff, and patient levels. Given this complexity, effective solutions are likely to call for multipronged interventions [6, 7]. In 2010, Uganda developed and piloted such an approach known as the Supervision, Performance Assessment, and Recognition Strategy (SPARS) which involved supportive supervision for health facility staff, systematic performance assessment using indicators during supervisory visits, sharing performance with managers at all levels through a pharmaceutical information system, and formal recognition of good performance including infrastructure improvements [7]. Behavior change interventions are more successful when they consist of a multipronged approach [8–11].

SPARS was implemented by district health workers trained as medicines management supervisors (MMS) who conducted SPARS visits along with their other duties. Each district has assigned one district-level MMS to oversee sub-district MMS and to supervise higher-level facilities (hospitals and health centers (HC)4) and two to five sub-district MMS per district supervise lower-level health centers (HC3 and HC2). The MMS assess performance in both government and private not-for-profit (PNFP) faith-based health facilities, discuss findings with health care staff, and agree on improvement targets at each visit. The Ministry of Health (MOH) manages SPARS implementation through an established unit in the pharmacy department, which is supported by regional pharmacists based at the regional hospital. Since SPARS inception, faculty staff from Makerere University have provided a 2-week training course for all MMS, which together with a package of standardized tools, manuals with standard operating procedures, job aids, and reimbursement for supervisory visits strengthens uniform program implementation. To simplify administration, all MMS are eligible for a standardized supervision fee of about US$12 to cover motorbike fuel, maintenance and a daily allowance when a SPARS report is submitted using the provided netbook and airtime. The MMS are not restricted to using the motorbikes and netbook only for their SPARS duties, which has encouraged them to take good care of the items [7].

MMS assess performance based on 25 indicators covering three supply chain and two AMU domains (prescribing and dispensing). The SPARS AMU indicators include most of the widely used WHO/International Network for Rational Use of Drugs indicators [12]. The SPARS method and indicators have been described elsewhere [7]. Prior to SPARS implementation, the average AMU domain scores assessed in 1384 facilities were 2.1 for dispensing quality and 0.9 for prescribing quality out of a maximum of 5.0, which illustrated the need to strengthen AMU at all levels of care [7]. This study reports on the impact of SPARS supervision on the measures that comprise the two domains of AMU indicators.

## Methods

### Design

This retrospective study assesses changes in AMU prescribing and dispensing measures from the first to the last supervisory visit during the first year of SPARS supervision in public and private not-for-profit health faith-based facilities at all levels of care in 45 districts in Uganda.

### Setting and context

Uganda had an estimated population of more than 40 million individuals in 2018 and an estimated annual growth rate of 3.2% per year [13]. Health care services were provided in the 89 districts that existed in 2010 through 6404 public and private sector health facilities, of which 3084 (48%) were government owned, 2373 (37%) were private, and 947 (15%) were PNFP facilities [14]. Uganda’s health facilities are divided into seven levels based on the services they provide and the catchment area they serve. The lowest HC1 level comprises village health teams, followed by increasingly larger HC2, HC3, and HC4 health centers; at the highest levels are general or district hospitals, regional referral hospitals, and the two national referral hospitals existing at the time [14]. Nurses primarily staff HC2, clinical officers and nurses’ staff HC3, and doctors, clinical officers, nurses, dispensers or pharmacy technicians, and storekeepers’ staff HC4 and hospitals. Nurses manage medicines at most health facilities because less than 8% of pharmacy posts in the public sector are filled; in general, pharmacists and pharmacy technicians are only available at higher-level facilities [15].

Essential medicines are provided free of charge to patients at government-owned health facilities; patients need to purchase medicines at PNFP facilities. Per capita expenditure on essential medicines in the public sector was US$2.40 in 2013/2014, of which US$0.99 was for basic essential medicines, and the remaining US$1.41 was for medicines to treat HIV, tuberculosis, and malaria. Public funding for essential medicines is inadequate to meet needs, and facilities are heavily dependent on donor funds, which covered 77% of essential medicine costs in 2013/2014 [15]; in addition, availability of essential medicines remains low at National Medical Stores—meeting 56–65% of need in public health facilities overall [16].

### Sampling

In 2009, we contacted district health officers from the then 80 districts in Uganda about interest in implementing SPARS. The overall response rate was 81% (*n* = 65/80). We ranked responsive districts according to their commitment to improving the availability of EMHS and scored their estimated capacity to carry out SPARS based on six evaluation criteria: district profile (size, population, number of facilities, Internet connectivity), infrastructure (district store size and condition), EMHS (availability and district distribution issues and solutions), partners (number and type of other development partners in the district), management and finance (per capita EMHS budget and expenditures), and staff (number of pharmaceutical staff members). Based on their scores, we classified their estimated capacity into “high,” “medium,” and “low” strata.

We randomly selected 44 districts from the three strata (high, medium, and low) using systematic sampling of 20, 12, and 12 districts, respectively, and checked that all four regions were equally represented; one more Western district was later selected randomly from all districts to reach a total of 45 districts, resulting in 15, 13, 9, and 8 districts from the Western, Eastern, Northern, and Central regions, respectively [7, 17]. This analysis includes 1222 SPARS supervised facilities from within the selected district that had at least two visits during a 12-month period of SPARS implementation. While the districts were randomly selected [7], the facilities were selected by the MMS with the aim to include all facilities in the selected district. We stratified the analysis by level of care but grouped HC4 and hospitals together due to the small sample size. We examined changes between the first and last SPARS visit during the year.

### Data sources and outcome variables

The SPARS performance assessment uses practical performance indicators to flag areas for improvement in a real-life setting, to guide and focus the supervision, and to provide health care staff with an understanding of their facility’s issues and achievements. The SPARS data collection tool has been detailed previously [7]. For this study, we examined the AMU indicators in the areas of dispensing quality (seven indicators) and prescribing quality (five indicators). Each indicator is assessed as a summary score based on a total of 25 and 32 individual dispensing and prescribing practices, respectively. To assess the AMU measures, the MMS review the facility dispensing and prescribing log and the laboratory log, observe staff practices, and interview patients as they exit the health facility to assess their knowledge and examine the labels on the medicines they received. The MMS records the findings in a structured data collection tool (Additional file 1) and submits them to a centralized electronic database. Additional file 2 lists the 57 AMU measures and their calculations. All measures are recorded on a binary scale of 1 (yes) or 0 (no); some values are then averaged across patients or records to obtain a summary score.

We assessed the impact of SPARS using 57 AMU measures evaluated during the first and last visits that occurred during the first year of follow-up in each facility. Our primary outcomes assessed change in percentage for 56 of the measures; indicator #33 (average number of medicines prescribed) was assessed as a mean score. We categorized the 57 AMU measures into two categories: 43 (75%) that can be improved primarily by behavior change on the part of the facility staff (“behavioral” or B) and 14 (25%) that require additional resources to be improved (“resource” or R). Given that SPARS is intended to provide attainable incentives for improvement, a SPARS score of 75% or more (18.25 on a possible 25 scale points) following 12 months of supervision was defined as “adequate” performance.

We assessed completeness of the data for each AMU measure in the first and last supervisory visit at each facility. The completeness of each of the individual measures during these visits is given in Additional file 3. Completeness was very high at both the initial and follow-up visits (over 90%) for nearly all measures, so we included all measures in the analysis.

### Predictor variables

The predictor variables used in this analysis have been described in a previous article in this series [17]. We identified two categories of predictor variables—facility-level and MMS-level predictors. We obtained facility variables from administrative data or SPARS visit records. We also linked AMU measures with results of a survey completed in 2013 by 111 (74.5%) of the 149 MMS represented in the study. The MMS variables were specific to the MMS who conducted the supervisory visit.

The facility-level predictors included facility ownership (government or PNFP), supervision structure, MMS qualifications, and district health officer (DHO) engagement (Tables 1, 2, 3, and 4). The supervision structure predictors included the number of SPARS visits in the initial year, the number of health facility staff supervised in the initial visit, the number of facilities assigned to the MMS conducting the visit, and whether the facility was supervised by one MMS alone or by two MMS supervising the facility together. Often the district MMS would mentor the other MMS in the district on their first supervisory visits or from time to time to assure uniformity in the SPARS implementation. Visits with two MMS could include both first and last visit to a health facility. The MMS predictors included gender, MMS position (district or sub-district supervisor), professional training (doctor/clinical officer, pharmacist/dispenser, nurse/midwife, stores officer), highest level of education, and years of work experience. MMS-specific indicators also included DHO engagement predictors (the frequency of MMS meeting with the DHO, whether the MMS received feedback from the DHO about reports), whether the MMS felt that there was sufficient time to provide adequate supportive supervision during a visit, and whether the MMS felt that health care workers responded well to the supervision. Based on data from complete cases, we used multiple methods to impute the values of missing survey predictors for use in multivariate regression models [18, 19].

**Table 1:**
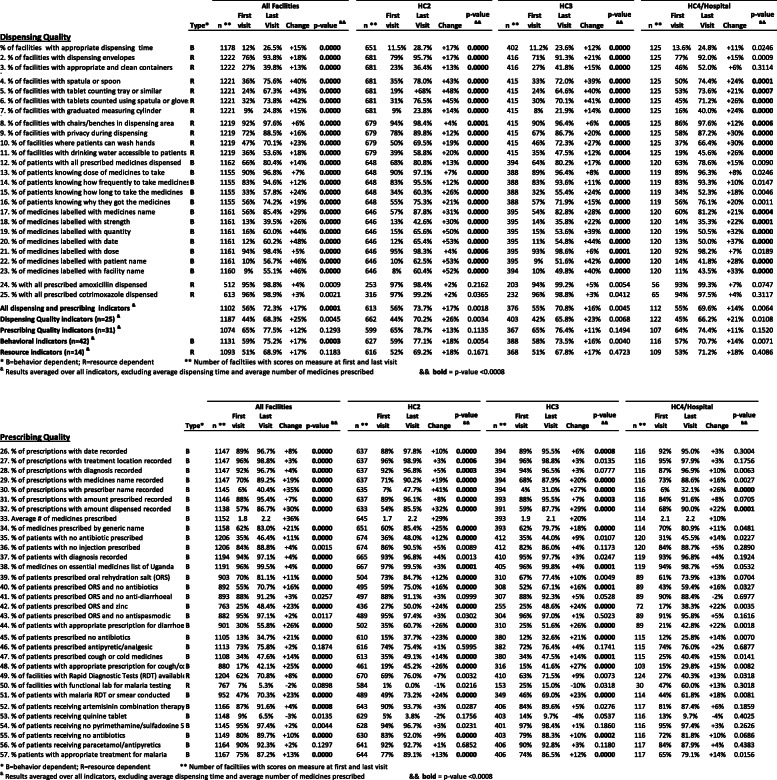
Average percentage point change in AMU measures: individually and by category and level of care

*B behavior dependent, *R* resource dependent

**Number of facilities with scores on measure at first and last visit

^&^Results averaged over all indicators, excluding average dispensing time and average number of medicines prescribed

^&&^Bold indicates *p* value < 0.0008

**Table 2:**
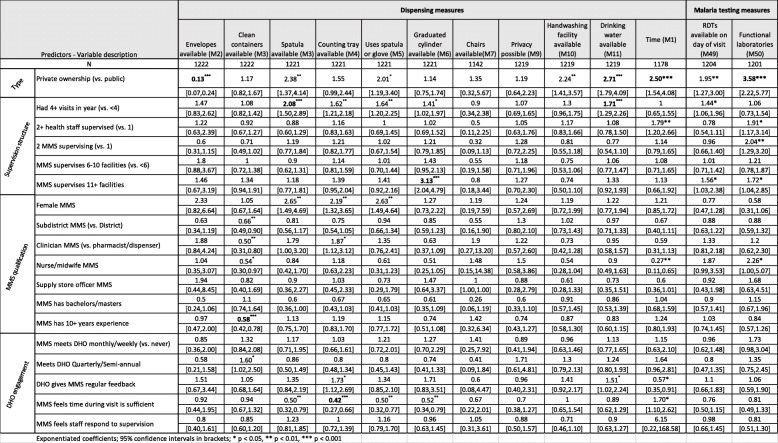
Predictors associated with AMU measures scored as yes/no at each facility in multivariate logistic models

Exponentiated coefficients; 95% confidence intervals in brackets

*p < 0.05, **p < 0.01, ***p < 0.001

**Table 3:**
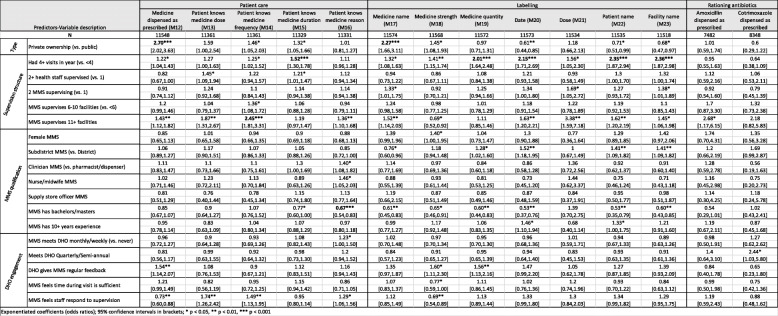
Predictors associated AMU measures scored as percentages in multivariate hierarchical logistic models

Exponentiated coefficients (odds ratios); 95% confidence intervals in brackets

*p < 0.05, **p < 0.01, ***p < 0.001

**Table 4:**
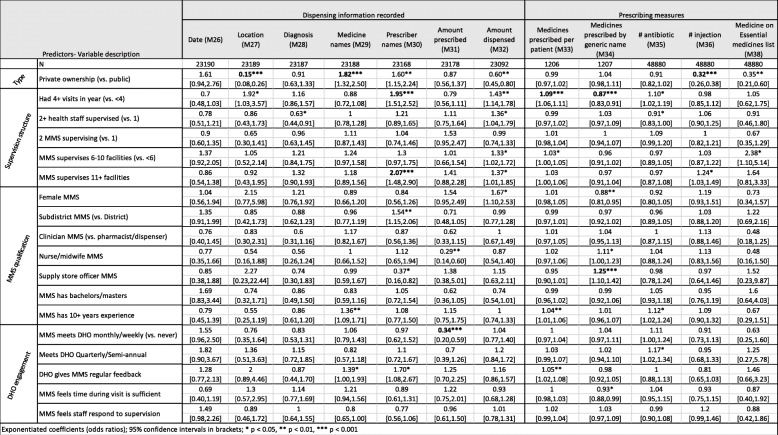
Predictors associated with dispensing and prescribing measures scored as percentages in multivariate hierarchical logistic models

Exponentiated coefficients (odds ratios); 95% confidence intervals in brackets

*p < 0.05, **p < 0.01, ***p < 0.001

### Statistical analysis

We first calculated each of the 57 AMU measures at first and last visits, as described in Additional file 1. Average percentages across facilities, both overall and by facility level, were calculated for all measures (except *average number of medicines prescribed*). We also calculated the percentage point change between visits for all facilities that had a measure at both the first visit and at the last visit. We used two sample tests of proportions (i.e., *Z*-tests) to test for statistically significant differences between the first and last visit. To avoid type 2 errors due to multiple testing, we used a Bonferroni correction (i.e., alpha = 0.05 divided by number of measures = 57). The *p* value threshold was therefore set at 0.0008 for statistical significance.

To assess the association between each predictor variable and the outcomes of interest, we used multivariable logistic regression models for outcomes with one score per facility (13 measures) and multivariate general linear regression models for outcomes with more than one score per facility (42 measures based on samples of patients, prescriptions, or medicines). Two indicators were not tested (diagnosis recorded (indicator 37) and oral rehydration solution (ORS) prescribed with no antispasmodics (indicator 43)), both with high initial implementation and thus minimal impact. We used proc logistic in SAS 9.3 to run the models and report the odds ratios (exponentiated coefficients) and 95% confidence intervals for each measure.

## Results

### Characteristics of health facilities and MMS

Between 2010 and 2013, 1222 health facilities starting the SPARS program had at least one follow-up visit in the next 12 months (Additional file 4). Of the 1222 facilities, 85% were government and 15% were PNFP facilities; 681 (56%), 416 (34%), and 125(10%) were HC2, HC3, and HC4/hospitals, respectively. The numbers of facilities by level of care were comparable across regions. Consistent with national staffing norms at lower and higher-level facilities, generally only one staff at lower-level health facilities was supervised by the MMS, compared with upper-level facilities where the MMS more often mentored two or more staff members.

A facility’s designated MMS conducted the initial supervision in about two thirds of facilities, while in the other facilities, the district MMS or another MMS not designated to that facility undertook the initial supervisory visit. MMS carried out 4172 supervisory visits in the first year of SPARS, with an average of 3.4 visits per facility and a median of 88 days between visits. The median number of visits per year per designated MMS was 28 (IQR 17–39).

Of the 148 MMS in the study, 84% were male, 64% were health sub-district-level MMS, 55% supervised 10 facilities or fewer, and 59% were trained as clinical officers (Additional file 5). Among the 111 MMS responding to the 2013 survey, 42% were age 36 to 45, 83% had a secondary- or diploma-level education, and 40% had fewer than 10 years of experience. Over half of the MMS met with their DHO at least once a month, and 85% reported receiving feedback from the DHO on their submitted reports. Two thirds of MMS reported that they felt there was enough time to supervise health staff and that the health workers responded well to the supervision.

The time to supervise a facility depended on access to data which improved with systems being put in place; the remoteness of the facility as the MMS had to include the time to reach the facility, supervise and return the same day, and access to the staff which depended on the patient load the day of visit. While the MMS assessed all indicators at each visit, they focused their supervision on selected indicators.

### Changes in AMU measures

**Fig. 1:**
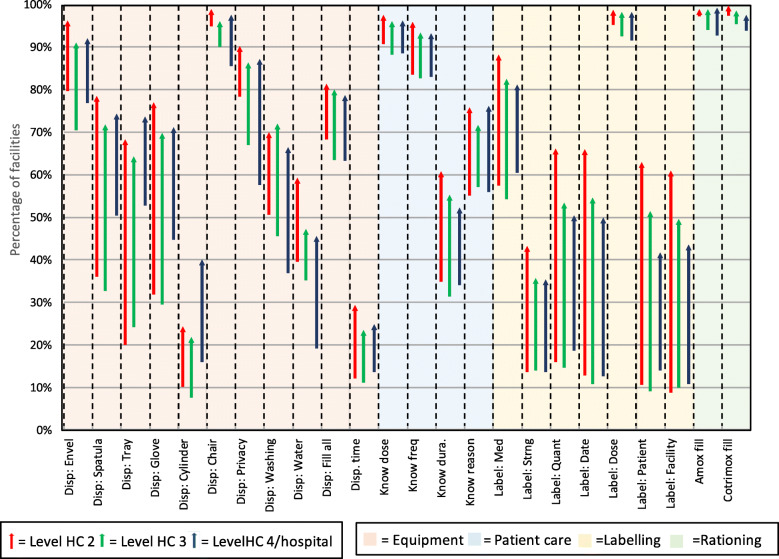
Changes in 24 dispensing measures from initial visit to last visit by level of care

**Fig. 2:**
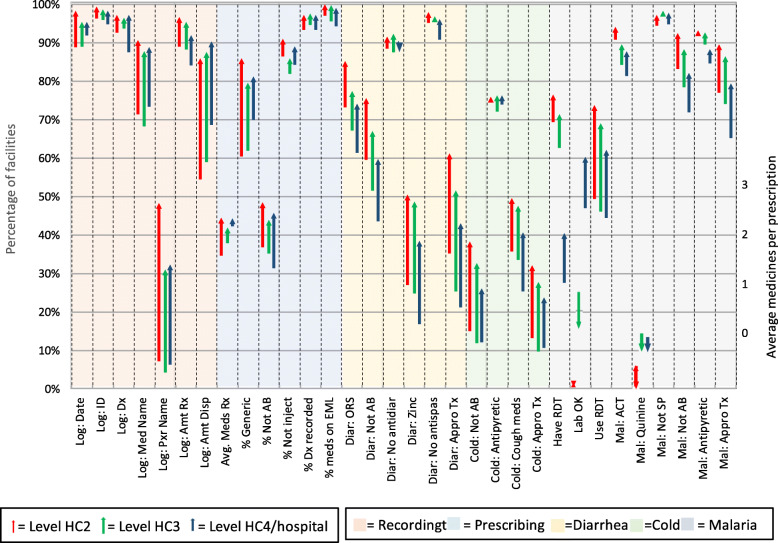
Changes in 32 prescribing measures from initial visit to last visit by level of care

Table 1 lists the values of the AMU individual, dispensing, overall and prescribing measures at the initial visit, changes from the initial to the last visit in the year, and the statistical significance of these changes by level of health care facility. Figure 1 illustrates the changes in the AMU measures during the year by level of care for the 24 dispensing measures and Fig. 2 illustrates changes in the 32 prescribing measures. Averaged over the 55 AMU measures based on percentages (excluding *average number of medicines per patient*), the overall average change during the year of supervision was a statistically significant improvement of 17 percentage points (25 and 12 percentage points for the dispensing and prescribing measures, respectively). Behavioral measures improved substantially at all levels of care; these changes were statistically significant in the overall sample; measures requiring additional resources improved by 17 percentage points, but this was not statistically significant.

### Dispensing measures

All but one of the dispensing quality measures showed significant improvements in the overall sample and all except two improved significantly in HC2 and HC3; the values of the measures that experienced nonsignificant improvements (*receiving all prescribed amoxicillin dispensed* and *receiving all prescribed cotrimoxazole dispensed*) averaged well over 90% at baseline, so had little room to improve. Changes in the dispensing measures in HC4/hospitals tended to be slightly smaller than those observed in lower-level facilities, and the final percentage point in the last visit also tended to be slightly lower; only 14 of the 25 measures at the highest level of care experienced changes that reached the designated level of statistical significance (Table 1).

Among the individual measures, the percentage of facilities with appropriate dispensing time of more than 60 s improved from 12 to 27% following 1 year of supervision, which was a significant improvement of 15 percentage points. The availability of dispensing equipment and services improved significantly (*p* < 0.0008) for all 11 measures across all levels of care, except for the availability of dispensing envelopes and clean containers in HC4/hospitals. The availability of dispensing envelopes stamped with fill-in directions for use improved by 18 percentage points overall, reaching 94% of all facilities by the end of the year. Use of spatulas, trays, and gloves for dispensing experienced some of the largest increases of all the dispensing quality measures in HC2 and HC3. The measure proving most resistant to change was availability of graduated cylinders, which only reached 25% of facilities by year’s end, although this still represented a significant improvement over the baseline of 15% (Table 1).

Patient knowledge of dose and frequency of administration of the medicines they received was high in all levels of care during the first visit, but nevertheless improved significantly during the year overall and in lower levels of care. We saw substantial improvements at all levels of care in patient knowledge about how long they were to take their medicines (+ 24 percentage points, *p* < 0.0008) and why they received them (+ 19 percentage points, *p* < 0.0008).

Appropriate labeling of dispensed medicines (i.e., writing the patient’s name, facility’s name, and medication’s strength, quantity, and dispensing date on the label) had low average baseline measures of less than 20% in all levels of care. Large and statistically significant improvements were observed for all these measures by the end of the year at all levels of care with improvements in the range of 21 to 53 percentage points.

Following 1 year of supervision, the labels on medicines that patients received included on average 65% of needed information measured through seven indicators and 81% of the patient information across four indicators required to best ensure appropriate use. (Table 1)

### Prescribing measures

Overall, health facilities experienced statistically significant improvements in 24 of the 31 prescribing measures during the year. Rates of improvement in HC2 and HC3 generally paralleled those in the overall sample, but fewer reached significance with 20 at HC2 and 16 at HC3; however, the HC4/hospitals had significant changes (*p* < 0.0008) in only two prescribing measures (Table 1). Measures for which no significant improvement was seen at any level of care (*no injection prescribed*, *ORS and no anti-diarrheal* and *ORS and no antispasmodic* prescribed for diarrhea, *receiving paracetamol/antipyretics* or *receiving no sulfadoxine/pyrimethamine* for malaria) had high practice performance scores at the initial visit and thus little room to improve. On the other hand, *prescribing of antipyretic or analgesics* for cough and cold and *availability of a functional laboratory* had low initial scores at all levels of care and did not experience significant improvements (Table 1).

At the first visit, most recommended practices for recording information in the prescribing or dispensing logs were already well followed, scoring 84% and above at all levels of care, except for recording the prescriber’s name (6% overall), the name of the medicine dispensed (70%), and the amount dispensed (57%). Large and statistically significant improvements were seen for all recording measures on average overall and for HC2 and at all levels of care in the recording of prescriber name and amount dispensed (Table 1).

The average number of medicines per prescription increased from 1.8 to 2.2 during the year of follow-up, which was a significant average increase of 22% for all levels of care; the increased averages were larger at lower levels of care (29%, 11%, and 5% for HC2, HC3, and HC4/hospitals, respectively). The rate of prescribing by generic name increased significantly at all levels of care, again with greater improvements at lower levels (25%, 18%, and 11% for HC2, HC3, and HC4/hospitals, respectively). Although the desired practice of not prescribing antibiotics during an encounter increased at all levels of care following supervision (reaching an overall average of close to half of all patients by the last visit), only the 12% improvement in HC2 was significant with *p* < 0.0008. The remaining prescribing measures were consistent with recommendations at the initial visit and thus experienced only small improvements.

Adherence to treatment recommended by the Uganda STGs [20] increased significantly for all three diagnoses assessed by the AMU measures—non-bloody diarrhea, mild upper respiratory tract infection (URTI) diagnosed as cough or cold, and malaria—with overall increases of 26, 25, and 13 percentage points, respectively, with gains statistically significant overall and at lower levels of care.

Of the AMU measures related to diarrheal treatment, use of oral rehydration solution (ORS) was relatively high at the initial visit in all levels of care (70%, 73%, and 67% for HC2, HC3, and HC4/hospitals, respectively), but nevertheless increased significantly by 11 percentage points during the year of supervision. The recommended practice of not prescribing antibiotics for watery diarrhea increased by 16 percentage points in all levels of care, increases that were significant overall and in lower-level facilities. Use of zinc as an adjunct treatment for diarrhea almost doubled from only 25% at first visit to 49% after a year of supervision.

Of patients assessed for treatment of cough or cold at the initial visit, only 13% did not receive antibiotics, which represented the worst practice of all the prescribing indicators. Improvements in practice were higher in HC2 and HC3 (a significant 23- and 21-percentage point increase, respectively) than in HC4/hospitals (14 percentage points). Despite these gains, about two thirds of the patients with cough or cold were still being prescribed antibiotics.

To ensure appropriate treatment of malaria, it is critical to initially perform a malaria diagnostic test and, if positive, to treat appropriately with an artemisinin-based combination therapy (ACT). While the availability of a functional laboratory decreased at lower levels of care during the year, the availability of rapid diagnostic tests (RDTs) increased significantly by 8 percentage points, reaching an overall availability of 71%, higher at HC2 and in HC2 and much less in HC4/hospitals. During the initial SPARS visits, only about half of patients at all levels of care were actually tested with RDTs; however, the use of RDTs for suspected malaria increased substantially by 23 percentage points, such that more than 70% of suspected cases were being tested by the end of the year. The other indicators assessing appropriate use of medicines for malaria treatment were high at the initial visit yet increased during the year.

### Achievement of adequate AMU performance

**Fig. 3:**
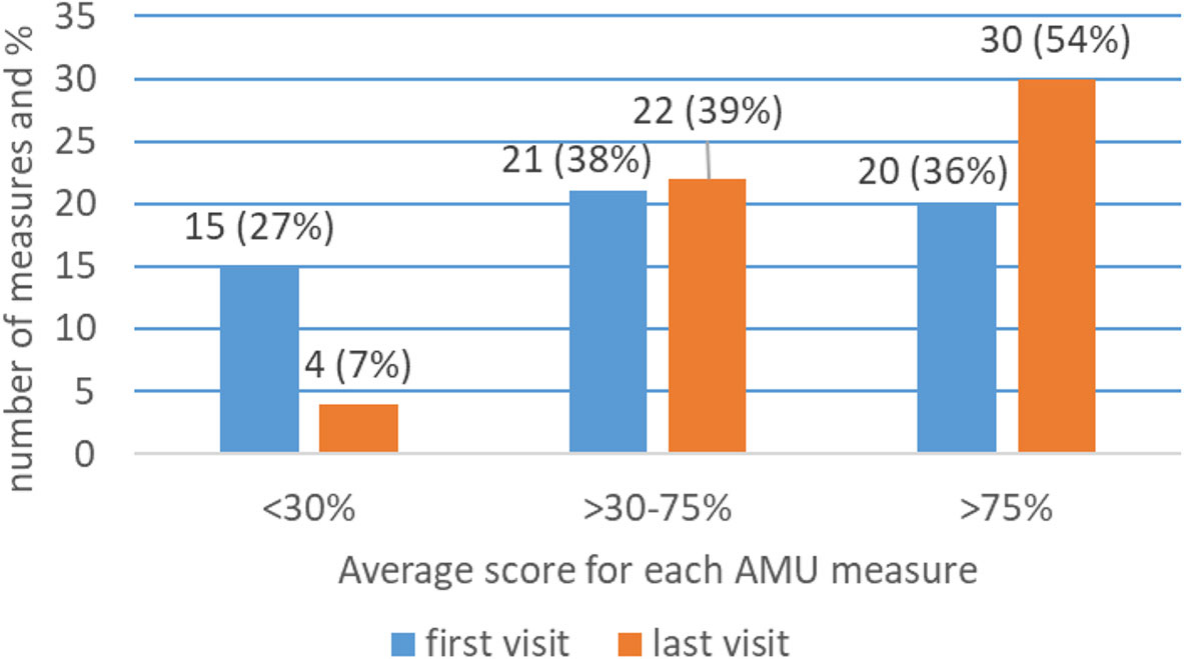
Average scores for 55 AMU percentage measures at first and last visit (*n* = 1222)

**Fig. 4:**
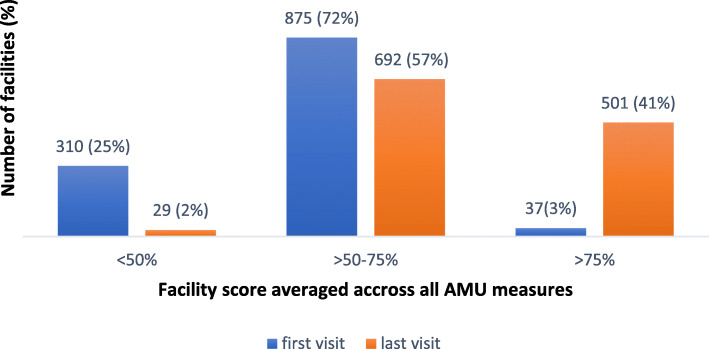
Average scores across all AMU measures at first and last visit for 1222 facilities

The distribution of the average scores for the 56 AMU percentage measures, excluding average number of medicines prescribed per patient, for the first and last visits are depicted in Figs. 3 and 4. The proportion of AMU measures for which facilities achieved an average acceptable score of 75% or above improved from 36% of measures at visit 1 to 55% at the last visit (*n* = 1222), an improvement which was consistent across levels of care (Fig. 3). At the facility-specific level (Fig. 4), only 3% of facilities achieved a positive average score of 75% or above on all measures at the initial visit, which increased to 41% of facilities following supervision.

### Predictors of change

We analyzed the predictors of improvement (i.e., final score adjusted for initial score) in the 13 SPARS measures that were scored as binary outcomes at each visit (Table 2), in the 14 dispensing measures scored as percentages of patients or medicines (Table 3), and in the prescribing measures scored as percentages of patients (Tables 4 and 5). Notable variations in improvement by key predictors included the following:

**Table 5:** Predictors associated with AMU treatment measures scored as percentages in multivariate hierarchical logistic models

	Type^a^	All facilities					HC2					HC3					HC4/Hospital				
		n^b^	First visit	Last Visit	Change	*p*-value ^&&^	n^b^	First visit	Last Visit	Change	*p*-value ^&&^	n^b^	First visit	Last Visit	Change	*p*-value ^&&^	n^b^	First visit	Last Visit	Change	*p*-value ^&&^
**Dispensing Quality**																					
% of facilities with appropriate dispensing time	**B**	1178	12%	26.5%	+15%	**0.0000**	651	11.5%	28.7%	+17%	**0.0000**	402	11.2%	23.6%	+12%	**0.0000**	125	13.6%	24.8%	+11%	0.0246
2. % of facilities with dispensing envelopes	**R**	1222	76%	93.8%	+18%	**0.0000**	681	79%	95.7%	+17%	**0.0000**	416	71%	91.3%	+21%	**0.0000**	125	77%	92.0%	+15%	0.0009
3. % of facilities with appropriate and clean containers	**R**	1222	27%	39.8%	+13%	**0.0000**	681	23%	36.4%	+13%	**0.0000**	416	27%	41.8%	+15%	**0.0000**	125	46%	52.0%	+6%	0.3114
4. % of facilities with spatula or spoon	**R**	1221	36%	75.6%	+40%	**0.0000**	681	35%	78.0%	+43%	**0.0000**	415	33%	72.0%	+39%	**0.0000**	125	50%	74.4%	+24%	**0.0001**
5. % of facilities with tablet counting tray or similar	**R**	1221	24%	67.3%	+43%	**0.0000**	681	19%	+68%	+48%	**0.0000**	415	24%	64.6%	+40%	**0.0000**	125	53%	73.6%	+21%	**0.0007**
6. % of facilities with tablets counted using spatula or gloves	**R**	1221	32%	73.8%	+42%	**0.0000**	681	31%	76.5%	+45%	**0.0000**	415	30%	70.1%	+41%	**0.0000**	125	45%	71.2%	+26%	**0.0000**
7. % of facilities with graduated measuring cylinder	**R**	1221	9%	24.8%	+15%	**0.0000**	681	9%	23.8%	+14%	**0.0000**	415	8%	21.9%	+14%	**0.0000**	125	16%	40.0%	+24%	**0.0000**
8. % of facilities with chairs/benches in dispensing area	**R**	1219	92%	97.6%	+6%	**0.0000**	679	94%	98.4%	+4%	**0.0001**	415	90%	96.4%	+6%	**0.0005**	125	86%	97.6%	+12%	**0.0006**
9. % of facilities with privacy during dispensing	**R**	1219	72%	88.5%	+16%	**0.0000**	679	78%	89.8%	+12%	**0.0000**	415	67%	86.7%	+20%	**0.0000**	125	58%	87.2%	+30%	**0.0000**
10. % of facilities where patients can wash hands	**R**	1219	47%	70.1%	+23%	**0.0000**	679	50%	69.5%	+19%	**0.0000**	415	46%	72.3%	+27%	**0.0000**	125	37%	66.4%	+30%	**0.0000**
11. % of facilities with drinking water accessible to patients	**R**	1219	36%	53.6%	+18%	**0.0000**	679	39%	58.8%	+20%	**0.0000**	415	35%	47.5%	+12%	**0.0004**	125	19%	45.6%	+26%	**0.0000**
12. % of patients with all prescribed medicines dispensed	**B**	1162	66%	80.4%	+14%	**0.0000**	648	68%	80.8%	+13%	**0.0000**	394	64%	80.2%	+17%	**0.0000**	120	63%	78.6%	+15%	0.0090
13. % of patients knowing dose of medicines to take	**B**	1155	90%	96.8%	+7%	**0.0000**	648	90%	97.1%	+7%	**0.0000**	388	89%	96.4%	+8%	**0.0000**	119	89%	96.3%	+8%	0.0246
14. % of patients knowing how frequently to take medicines	**B**	1155	83%	94.6%	+12%	**0.0000**	648	83%	95.5%	+12%	**0.0000**	388	83%	93.6%	+11%	**0.0000**	119	83%	93.3%	+10%	0.0147
15. % of patients knowing how long to take the medicines	**B**	1155	33%	57.8%	+24%	**0.0000**	648	34%	60.3%	+26%	**0.0000**	388	32%	55.4%	+24%	**0.0000**	119	34%	52.3%	+18%	0.0046
16. % of patients knowing why they got the medicines	**B**	1155	56%	74.2%	+19%	**0.0000**	648	55%	75.3%	+21%	**0.0000**	388	57%	71.9%	+15%	**0.0000**	119	56%	76.1%	+20%	0.0011
17. % of medicines labelled with medicines name	**B**	1161	56%	85.4%	+29%	**0.0000**	646	57%	87.8%	+31%	**0.0000**	395	54%	82.8%	+28%	**0.0000**	120	60%	81.2%	+21%	**0.0004**
18. % of medicines labelled with strength	**B**	1161	13%	39.5%	+26%	**0.0000**	646	13%	42.6%	+30%	**0.0000**	395	14%	35.8%	+22%	**0.0000**	120	14%	35.3%	+22%	**0.0001**
19. % of medicines labelled with quantity	**B**	1161	16%	60.0%	+44%	**0.0000**	646	15%	65.6%	+50%	**0.0000**	395	15%	53.6%	+39%	**0.0000**	120	19%	50.5%	+32%	**0.0000**
20. % of medicines labelled with date	**B**	1161	12%	60.2%	+48%	**0.0000**	646	12%	65.4%	+53%	**0.0000**	395	11%	54.8%	+44%	**0.0000**	120	13%	50.0%	+37%	**0.0000**
21. % of medicines labelled with dose	**B**	1161	94%	98.4%	+5%	**0.0000**	646	95%	98.3%	+4%	**0.0006**	395	93%	98.6%	+6%	**0.0001**	120	92%	98.2%	+7%	0.0189
22. % of medicines labelled with patient name	**B**	1161	10%	56.7%	+46%	**0.0000**	646	10%	62.5%	+53%	**0.0000**	395	9%	51.6%	+42%	**0.0000**	120	14%	41.8%	+28%	**0.0000**
23. % of medicines labelled with facility name	**B**	1160	9%	55.1%	+46%	**0.0000**	646	8%	60.4%	+52%	**0.0000**	394	10%	49.8%	+40%	**0.0000**	120	11%	43.5%	+33%	**0.0000**
24. % with all prescribed amoxicillin dispensed	**R**	512	95%	98.8%	+4%	0.0009	253	97%	98.4%	+2%	0.2162	203	94%	99.2%	+5%	0.0054	56	93%	99.3%	+7%	0.0747
25. % with all prescribed cotrimoxazole dispensed	**R**	613	96%	98.9%	+3%	0.0021	316	97%	99.2%	+2%	0.0365	232	96%	98.8%	+3%	0.0412	65	94%	97.5%	+4%	0.3117
**Prescribing Quality**																					
26. % of prescriptions with date recorded	**B**	1147	89%	96.7%	+8%	**0.0000**	637	88%	97.8%	+10%	**0.0000**	394	89%	95.5%	+6%	**0.0008**	116	92%	95.0%	+3%	0.3004
27. % of prescriptions with treatment location recorded	**B**	1147	96%	98.8%	+3%	**0.0000**	637	96%	98.9%	+3%	**0.0006**	394	96%	98.8%	+3%	0.0135	116	95%	97.9%	+3%	0.1756
28. % of prescriptions with diagnosis recorded	**B**	1147	92%	96.7%	+4%	**0.0000**	637	92%	96.8%	+5%	**0.0003**	394	94%	96.5%	+3%	0.0777	116	87%	96.9%	+10%	0.0063
29. % of prescriptions with medicines name recorded	**B**	1147	70%	89.2%	+19%	**0.0000**	637	71%	90.2%	+19%	**0.0000**	394	68%	87.9%	+20%	**0.0000**	116	73%	88.6%	+16%	0.0027
30. % of prescriptions with prescriber name recorded	**B**	1145	6%	40.4%	+35%	**0.0000**	635	7%	47.7%	+41%	**0.0000**	394	4%	31.0%	+27%	**0.0000**	116	6%	32.1%	+26%	**0.0000**
31. % of prescriptions with amount prescribed recorded	**B**	1146	88%	95.4%	+7%	**0.0000**	637	89%	96.1%	+8%	**0.0000**	393	88%	95.5%	+7%	**0.0003**	116	84%	91.6%	+8%	0.0705
32. % of prescriptions with amount dispensed recorded	**B**	1138	57%	86.7%	+30%	**0.0000**	633	54%	85.5%	+32%	**0.0000**	391	59%	87.7%	+29%	**0.0000**	114	68%	90.0%	+22%	**0.0001**
33. Average # of medicines prescribed	**B**	1152	1.8	2.2	+36%		645	1.7	2.2	+29%		393	1.9	2.1	+20%		114	2.1	2.2	+10%	
34. % of medicines prescribed by generic name	**B**	1158	62%	83.0%	+21%	**0.0000**	651	60%	85.4%	+25%	**0.0000**	393	62%	79.7%	+18%	**0.0000**	114	70%	80.9%	+11%	0.0481
35. % of patients with no antibiotic prescribed	**B**	1206	35%	46.4%	+11%	**0.0000**	674	36%	48.0%	+12%	**0.0000**	412	35%	44.0%	+9%	0.0107	120	31%	45.5%	+14%	0.0227
36. % of patients with no injection prescribed	**B**	1206	84%	88.8%	+4%	0.0015	674	86%	90.5%	+5%	0.0089	412	82%	86.0%	+4%	0.1173	120	84%	88.7%	+5%	0.2890
37. % of patients with diagnosis recorded	**B**	1194	94%	97.1%	+4%	**0.0000**	665	93%	96.8%	+4%	0.0013	410	95%	97.7%	+3%	0.0247	119	93%	96.8%	+4%	0.1924
38. % of medicines on essential medicines list of Uganda	**B**	1191	96%	99.5%	+4%	**0.0000**	667	97%	99.5%	+3%	**0.0001**	405	96%	99.8%	+4%	**0.0001**	119	94%	98.7%	+5%	0.0532
39. % of patients prescribed oral rehydration salt (ORS)	**B**	903	70%	81.1%	+11%	**0.0000**	504	73%	84.7%	+12%	**0.0000**	310	67%	77.4%	+10%	0.0049	89	61%	73.9%	+13%	0.0704
40. % of patients prescribed ORS and no antibiotics	**B**	892	55%	70.7%	+16%	**0.0000**	495	59%	75.0%	+16%	**0.0000**	308	52%	67.1%	+16%	**0.0001**	89	43%	59.4%	+16%	0.0327
41. % of patients prescribed ORS and no anti-diarrhoeal	**B**	893	88%	91.2%	+3%	0.0257	497	88%	91.1%	+3%	0.0999	307	88%	92.3%	+5%	0.0528	89	90%	88.4%	-2%	0.6977
42. % of patients prescribed ORS and zinc	**B**	763	25%	48.4%	+23%	**0.0000**	436	27%	50.0%	+24%	**0.0000**	255	25%	48.6%	+24%	**0.0000**	72	17%	38.3%	+22%	0.0035
43. % of patients prescribed ORS and no antispasmodic	**B**	882	95%	97.1%	+2%	0.0117	489	95%	97.4%	+3%	0.0302	304	96%	97.0%	+1%	0.5023	89	91%	95.8%	+5%	0.1616
44. % of patients with appropriate prescription for diarrhoea	**B**	901	30%	55.8%	+26%	**0.0000**	502	35%	60.7%	+26%	**0.0000**	310	25%	51.6%	+26%	**0.0000**	89	21%	42.8%	+22%	0.0018
45. % of patients prescribed no antibiotics	**B**	1105	13%	34.7%	+21%	**0.0000**	610	15%	37.7%	+23%	**0.0000**	380	12%	32.6%	+21%	**0.0000**	115	12%	25.8%	+14%	0.0070
46. % of patients prescribed antipyretic/analgesic	**B**	1113	73%	75.8%	+2%	0.1874	616	74%	75.4%	+1%	0.5995	382	72%	76.4%	+4%	0.1741	115	74%	76.0%	+2%	0.6877
47. % of patients prescribed cough or cold medicines	**B**	1108	34%	47.6%	+14%	**0.0000**	613	35%	49.1%	+14%	**0.0000**	380	34%	47.5%	+14%	**0.0001**	115	25%	40.4%	+15%	0.0141
48. % of patients with appropriate prescription for cough/cold	**B**	880	17%	42.1%	+25%	**0.0000**	461	19%	45.2%	+26%	**0.0000**	316	15%	41.6%	+27%	**0.0000**	103	15%	29.8%	+15%	0.0082
49. % of facilities with Rapid Diagnostic Tests (RDT) available	**R**	1204	62%	70.8%	+8%	**0.0000**	670	69%	76.0%	+7%	0.0032	410	63%	71.5%	+9%	0.0073	124	27%	40.3%	+13%	0.0318
50. % of facilities with functional lab for malaria testing	**R**	767	7%	5.3%	-2%	0.0898	584	1%	0.0%	-1%	0.0216	153	25%	15.0%	-10%	0.0318	30	47%	60.0%	+13%	0.3018
51. % of patients with malaria RDT or smear conducted	**B**	952	47%	70.3%	+23%	**0.0000**	489	49%	73.2%	+24%	**0.0000**	349	46%	69.0%	+23%	**0.0000**	114	44%	61.8%	+18%	0.0081
52. % of patients receiving artemisinin combination therapy	**B**	1166	87%	91.6%	+4%	**0.0008**	643	90%	93.7%	+3%	0.0287	406	84%	89.6%	+5%	0.0276	117	81%	87.4%	+6%	0.1859
53. % of patients receiving quinine tablet	**B**	1148	9%	6.5%	-3%	0.0135	629	5%	3.8%	-2%	0.1756	403	14%	9.7%	-4%	0.0537	116	13%	9.7%	-4%	0.4025
54. % of patients receiving no pyrimethamine/sulfadoxine SP	**B**	1145	95%	97.4%	+2%	0.0044	628	94%	96.7%	+3%	0.0231	401	97%	98.4%	+1%	0.1860	116	95%	97.4%	+3%	0.2626
55. % of patients receiving no antibiotics	**B**	1149	80%	89.7%	+10%	**0.0000**	630	83%	92.0%	+9%	**0.0000**	403	79%	88.3%	+10%	**0.0002**	116	72%	81.8%	+10%	0.0686
56. % of patients receiving paracetamol/antipyretics	**B**	1164	90%	92.3%	+2%	0.1297	641	92%	92.7%	+1%	0.6852	406	90%	92.8%	+3%	0.1180	117	84%	87.9%	+4%	0.4383
57. % patients with appropriate treatment for malaria	**B**	1167	75%	87.2%	+13%	**0.0000**	644	77%	89.1%	+13%	**0.0000**	406	74%	86.5%	+12%	**0.0000**	117	65%	79.1%	+14%	0.0156
**All indicators**^**c**^		1102	56%	72.3%	+17%	**0.0001**	613	56%	73.7%	+17%	0.0018	376	55%	70.8%	+16%	0.0045	112	55%	69.6%	+14%	0.0064
**Dispensing Quality indicators (*n*=25)**^**c**^		1187	44%	68.3%	+25%	0.0045	662	44%	70.2%	+26%	0.0034	403	42%	65.8%	+23%	0.0068	122	45%	66.2%	+21%	0.0108
**Prescribing Quality indicators (*n*=31)**^**c**^		1074	65%	77.5%	+12%	0.1293	599	65%	78.7%	+13%	0.1135	367	65%	76.4%	+11%	0.1494	107	64%	74.4%	+11%	0.1520
**Behavioral indicators (*n*=42)**^**c**^	**B**	1131	59%	75.2%	+17%	**0.0003**	627	59%	77.1%	+18%	0.0054	388	58%	73.5%	+16%	0.0040	116	57%	70.7%	+14%	0.0071
**Resource indicators (*n*=14)**^**c**^	**R**	1093	51%	68.9%	+17%	0.1183	616	52%	69.2%	+18%	0.1671	368	51%	67.8%	+17%	0.4723	109	53%	71.2%	+18%	0.4086

^&&^**bold** = *p*-value <0.0008

^a^*B* behavior dependent, *R* resource dependent

^b^Number of faciltiies with scores on measure at first and last visit

^c^Results averaged over all indicators, excluding average dispensing time and average number of medicines prescribed

#### Ownership (PNFP versus government owned)

Several AMU measures improved significantly more in PNFP facilities compared to government facilities following SPARS supervision, including increased availability of drinking water (odds ratio = 2.71), better practices in writing the name of the medicines dispensed on the label (2.27), increased dispensing time (2.50), and dispensing of the medicines that were prescribed (2.70). In the area of prescribing, PNFP facilities experienced larger improvements in comparison to public sector facilities in recording the medicines’ name (1.82), injection use (0.32), malaria testing (2.19), availability of functional laboratories for malaria testing (3.58), and appropriate use of quinine for severe malaria (6.61).

However, public sector facilities also experienced greater improvements in several measures. Compared to PNFP facilities, public sector facilities experienced greater improvements in availability of dispensing envelopes (0.13), increased use of ORS (0.54), appropriate treatment of diarrhea (0.60); ACT prescribing (0.22), not prescribing antibiotics in treatment of malaria (0.49), and appropriate malaria treatment (0.48).

#### Supervision structure

We found that having a greater number of visits in the first year was associated with greater improvements in availability of spatulas and drinking water and more patients knowing how long to take their medicines. Labeling practices also improved more in facilities that received more than four supervisory visits in the first year, including recording information on the quantity dispensed (2.01), date (2.15), patient name (2.35), and facility name (2.36); recording prescriber name in the dispensing log (1.95) also improved more in these facilities. Surprisingly, more frequent supervision was associated with an increased number of medicines prescribed per patient (1.09) and decreased use of generic prescribing (0.87). Greater improvements in testing for malaria (1.51) were observed when more than one staff member was supervised at a facility.

#### MMS qualifications

Greater improvements in treating diarrhea without antibiotics (0.60) were observed in r facilities supervised by female MMS and MMS educated as nurses or midwifes compared to MMS trained as other carders were less effective in improving diarrhea treatment without antibiotics. Being supervised by an MMS trained as a supply stores manager was associated with greater improvements in generic prescribing (1.25).

## Discussion

With an overall average 17-percentage point increase in AMU measures during the first year of supervision, representing a relative improvement of 30% from the initial visit, our study documents that SPARS is an effective multicomponent intervention to improve AMU at all levels of health care in both government and PNFP sectors. The overall performance improvement was twice as large for measures of appropriate dispensing (24 percentage points) than for measures of appropriate prescribing (12 percentage points), although the initial values for the dispensing measures were substantially lower (44% vs. 65%) and thus had greater range for improvement.

### Dispensing measures

On average, dispensing measures requiring additional resources, such as equipment, improved to the same degree as those requiring behavior change only. The development partner implementing the SPARS program was able provide needed resources such as dispensing envelopes, empty containers, measuring cylinders, and counting trays and assisted the Uganda government in national quantification exercises to ensure availability of medicines and RDTs. Assuring the availability of those resources is essential to maintain practice improvement. Making drinking water and hand-washing facilities available requires investment by districts or facilities, and about 20% of facilities were able to provide these resources during the year of supervision. Despite improvements, availability of graduated measuring cylinders and clean empty containers remained low during the follow-up year. Health facilities have shifted to dispensing mixtures in prefabricated bottles, so measuring cylinders and empty containers may no longer be needed in most facilities, although they remain a legal requirement.

Improvements in patient knowledge about the medicines dispensed and in labeling are closely linked to availability and use of dispensing envelopes, which increased from 76 to 94% of patient encounters. In addition to being more available, filling the label with correct information also improved significantly. The most common information given to patients prior to supervision covered how much to take and when, but other information such as duration was provided to only one third of patients; this patient care measure experienced the greatest improvement.

Poor availability of medicines and high out-of-pocket costs can result in not all prescribed medicines being dispensed. In the Ugandan context, the availability of six tracer medicines at health facility level increased from 61 to 88% from 2010/2011 to 2012/2013 following improved stock and storage management and increased per capita expenditures on essential medicines [15, 17]. Furthermore, medicines are provided free of charge in the government sector and some medicines are also donated in the PNFP sector. Thus, rationing of medicines was not found to be a major issue in this study. MMS advise facility staff to refer patients to another facility if medicines prescribed are not available, but the SPARS methodology does not measure the frequency of referrals, so it is unclear if this also increased during the year of supervision.

### Prescribing measures

To assess the adequacy of prescription recording, we measured the percent of entries in the dispensing or prescribing log that contained the information recommended for adequate documentation. Apart from name of the prescriber and the amount dispensed, most information was well recorded, even at the initial visit. In hospitals with many prescribers, names might not always be known or legible; at lower-level facilities with a single prescriber, it might be irrelevant to record the name.

Many of the WHO core prescribing indicators were in the range of adequate performance at the initial visit, including high rates of recording diagnosis, prescribing from the national essential medicines list, and limiting the use of injections. The number of medicines prescribed per patient increased from 1.8 to 2.2 following SPARS supervision, which is below the median of 2.8 medicines prescribed per patient found in 19 studies from 1990 to 2009 [4]. The increase may be linked in part to increased government funding for medicines during this period. Per capita expenditure on essential medicines and health supplies (excluding antiretrovirals, ACTs, tuberculosis supplies, and vaccines) doubled from $0.5 to almost $1 between 2010 and 2014 [15]. Of the core prescribing measures, we saw the largest increases in prescribing by generic name, reaching 83% by the end of the year. Overall use of antibiotics decreased by 11 percentage points, although 54% of patients were still prescribed antibiotics. These levels are similar to findings reported in other studies [4, 21, 22].

This study examined adherence to STGs for three common conditions: diarrhea, mild-to-moderate URTI, and malaria. A WHO review of published studies in low-income countries from 1990 to 2006 found that adherence to STGs remained unchanged at less than 40% [23]. In this study, we found wide variation in adherence to STGs by diagnosis prior to the SPARS intervention, with three fourths of malaria patients treated as recommended, compared to less than one third of patients with diarrhea and one fifth of those with cough and cold. For all three conditions, the primary inappropriate prescribing practice was overuse of antibiotics.

Diarrhea must be treated with ORS and optimally zinc, vitamin A, and medicines for deworming, but not antibiotics, antidiarrheal medicines, or antispasmodics. After 1 year of SPARS supervision, inappropriate use of antibiotics was reduced by one third (from 45 to 29%), although there was still ample opportunity for improvement. A previous study to improve treatment of acute diarrhea found a similar 17-percentage point reduction in inappropriate antimicrobial usage after a small-group face-to-face intervention [24].

Similarly, treatment of mild-to-moderate URTI does not require antibiotics, but rather symptomatic treatment with antipyretics, analgesics, or treatment of cough and cold. Following a year of supervision, inappropriate antibiotic use was reduced by more than 20 percentage points, but almost 70% of all patients with URTI still received antibiotics. The diagnosis recorded in most cases did not specify a mild, moderate, or severe infection, so our sample may have included severe cases, where antibiotic treatment can be justified; however, in Uganda, we estimate that 20% or less of cases are severe, suggesting that most cases would not require antibiotic treatment. We observed a lower percentage of antibiotic use at lower levels of care. Lower-level facilities typically are staffed by nurses or nurse aides who have been found to follow guidelines more than doctors [4]. In addition, supervision at lower-level facilities is provided more frequently to the same person(s) and therefore may be more effective. Moreover, the services provided and antibiotics available are simpler and fewer compared to higher levels of care. These factors may encourage more guideline-adherent practices in treating URTI.

Testing for malaria prior to treatment is a strategy to improve case management of fever and better targeting of treatment with ACTs. HC2 and most HC3 are expected to use RDTs for testing, while higher-level facilities are expected to have a laboratory to conduct malaria tests using microscopic analysis. Unfortunately, many laboratories were not well functioning due to lack of equipment, reagents, or human resources, so RDT is the primary testing method at higher-level facilities as well. RDTs are donor funded and should be fully available, supplied in kits at HC2 and HC3 and through regular orders at HC4/hospitals. Availability of RDTs increased significantly at all levels of care, although only three fourths of lower-level facilities had RDTs available on the day of the final supervisory visit. Most higher-level facilities were able to perform malaria testing, since 40% had RDTs available and 60% had a functioning laboratory. Following a year of SPARS supervision, 70% of patients diagnosed with malaria were tested for malaria. To further increase rates of guideline-adherent treatment, it is critically important to ensure both 100% availability of RDTs or functioning laboratory services and to monitor adherence to test results, which has been shown to be a major concern [25].

Standard treatment for malaria cases requires ACT or quinine; paracetamol is optional, while malaria cases should not be given antibiotics, sulfadoxine/pyrimethamine, or other medicines. After a year, rates of appropriate malaria treatment reached almost 90%, and most importantly, inappropriate use of antibiotics had been cut in half to about 10% of patients. Although we assessed if ACTs were prescribed, we were unable to look into patient adherence to treatment, which is a well-documented problem related to patient knowledge and information provided [26]. SPARS supervision emphasizes providing correct information to patients, a measure that significantly improved during the year.

Other studies of AMU interventions in low-resource settings suggest that supervision produces a small positive effect [8, 9, 27–29], but often, the studies did not use comprehensive methods to assess the full range of effects. The 17-percentage point overall AMU improvement associated with SPARS is consistent with results observed in interventions involving training and supervision, as recently reported in a large systematic review of strategies to improve health worker performance [11]. Another review of studies specifically related to medicine use found an average improvement of 10 to 12% in prescribing, which is again consistent with the 12-percentage point improvement in prescribing measures that we observed [4, 23]. The higher impact we observed in dispensing quality measures might be because these measures are easier to observe during a supervisory visit or that behaviors such as drug labeling are easier to change than prescribing [9].

### Predictor variables

A Cochrane review of 49 comparative intervention studies found that audit and feedback interventions are more effective when baseline performance is low and when feedback is provided by a “supervisor or senior colleague” and delivered at least “monthly,” in both a “verbal and written” format [29]. Our findings are consistent in that improvements are related to initial score, MMS education, and frequency of supervisory visits.

#### Facility ownership

Improvements in dispensing and prescribing measures varied between PNFP facilities and government facilities. PNFP facilities have to purchase dispensing envelopes, while the SPARS program provided them in public facilities. Higher rates of recording medicines’ names in PNFP facilities could be linked to the fact that patients have to buy medicines at PNFP facilities. PNFP facilities dispensed more of the medicines prescribed, which could be related to the fact that they order medicines based on need instead of depending on a kit supply with set quantities, as in lower-level government facilities; in addition, the PNFP medicines budget includes government funding combined with patient cost sharing.

Prescribing in PNFP facilities tended to be worse than in the public sector, with higher use of injections, less improvement in compliance to STGs for diarrhea and malaria, and more inappropriate prescribing of antibiotics for malaria treatment. Other studies have also shown that prescribing in the public sector was better than in the private sector [4, 23, 30]. However, mission-based health facilities are often better equipped with more functioning laboratories, which may explain why PNFP facilities tested for malaria more consistently. Poor adherence to guidelines is a serious concern because a major proportion of the population is served by the PNFP sector. Since 2013, the Ministry of Health has trained MMS from the four medical bureaus that comprise the PNFP sector to oversee and supervise PNFP facilities, shifting supervisory responsibility from government-employed MMS. The SPARS results feed into a pharmaceutical Information portal that now has a separate PNFP information and reporting system which can be used to monitor medicine use in PNFP.

#### Number of visits

Facilities having four or more supervisory visits experienced greater improvement in labeling. Good labeling requires dispensing envelopes and appropriate information recorded on the label. After dispensing envelopes are made available, it is not surprising that labeling improved with a greater number of reminders about appropriate practices, which has been found in other studies [29].

Having more than four MMS visits was associated with greater reductions in the average number of medicines prescribed per encounter, which may be a result of the strengthened adherence to standard treatment guidelines.

#### Level of care

SPARS supervision resulted in significant performance improvement at all levels of care despite differences in service complexity and staffing. Similar to other studies, we found that intervention effects varied by level of care [4, 8]. HC2 and HC3 facilities have fewer staff members, so supervision at those levels of care is more consistently provided one-on-one; moreover, the services provided are simpler and fewer compared to higher levels of care, which may be why we saw the greatest improvements in the measures of appropriate treatment for diarrhea, cough/cold, and malaria at lower levels of care. Differential increases in the number of medicines prescribed per encounter may be linked to referral of sicker patients to higher levels of care or greater access to more second- and third-line medicines. At higher-level facilities with several prescribers, it is critically important that all prescribers are supervised in order to sustain improvement.

### MMS characteristics

To be effective, the supervisor must be motivated, interested in being part of SPARS, and provide supportive supervision [31]. One could expect that an MMS with a clinical background would be better in changing prescribing practices and strengthening appropriate medicine use, while MMS trained in pharmaceuticals (i.e., stores managers, pharmacy technicians and pharmacists) would be better in strengthening dispensing quality. Our findings confirmed this only to some extent. Storekeepers trained as MMS had a greater impact on rates of generic prescribing in the facilities they supervised, presumably because they were very aware of the generic names of medicines from the medicines supply system in Uganda.

We only saw a gender difference related to one measure: the facilities with female MMS adhered better to STGs for diarrhea with fewer antibiotics prescribed. The explanation to this is not known. However, most health care staff are female and perhaps they viewed female supervisors as allies who understand the challenges in appropriate prescribing, so were more likely to comply with their recommendations.

### Limitations

The 45 districts were randomly selected for the study from the 89 districts that existed in 2010, representing a range of diversity, regional representation, poverty, and need and district performance classified as high, medium, or low, [7]. The district sample provides a good cross-sectional representation of Uganda; however, the selection of facilities is possibly biased because they were chosen by the MMS. The MMS were tasked to include all facilities in their district, but the order of inclusion was left to the MMS and the MMS may or may not have selected the facilities based on ownership, level of care, needs, and proximity as planned. Depending on the number of facilities allocated to the MMS, their time available and enthusiasm for supervision would differ; some MMS reached all their allocated facilities within the first year, while others met their targets only after more years of SPARS implementation. The supervised facilities represent about 30% of all government and PNFP facilities in the country; 85% of the sample facilities belonged to the government, compared to the actual proportion of 77% government facilities in the country [14]. The proportion of facilities to be included at each level of care was not specified in advance but determined separately in each district by the DHO and MMS.

The district leadership selected the MMS; although DHO received guidelines for MMS selection, considerable differences were observed in MMS experience, education, interest and willingness, supervisory skills, and the support given to MMS in different districts. Being a real-life field study, we did not try to control for these factors, but they likely influenced impact [31].

We followed each facility for 1 year from the date of the initial SPARS visit. However, the number of supervisory visits at each facility within that period varied from two to seven, with almost equal numbers of facilities having two, three, four, and five visits within the 12 months. Only 3% of the facilities had more than five visits and the average was 3.4 visits per facility. The number of visits to a facility depends on the ability of the MMS to allocate time and resources to implement SPARS. To ensure that transport did not cause a barrier, the implementing partner provided motorbikes and fuel. However, the SPARS supervision was an addition to the MMS’s other tasks, so existing workload might have had considerable impact on the number of monthly supervisory visits and on improvements observed.

Another important limitation to the study was the lack of a control group. We did not have access to a control group of a comparable size outside of the SPARS districts that was followed for 12 months. We believe that the consistent improvements in AMU performance we observed were likely due more to the intervention than any unobserved factors, but only better controlled or longer-term studies would be able to demonstrate that.

The analysis of predictors was limited by the 75% response rate from the MMS who completed the survey, despite numerous reminders and follow-up telephone calls. However, we used multiple imputation methods to impute values of missing survey predictors for use in regression models, and we found that results using only cases with complete data were basically equivalent to those obtained using imputed data.

Our study examined a wide range of outcomes and multiple testing might result in overestimation of significance due to the fact that 5% of comparisons will be significantly different by chance alone. To minimize the bias due to multiple testing, we used an adjusted significance level of < 0.001.

A greater number of facilities had initial visits in 2011 compared to 2012 and 2013. Facilities initiated later in the study or later within a district might have been better oriented on SPARS prior to initial visits, and MMS might have gained valuable experience from their earlier supervision experience. Although we cannot rule out the possibility that later initiation might have had an impact on the level of improvement observed, the size of observed changes generally did not differ by year of initiation. Also, several other programs such as the USAID funded STRIDES for family health, STAR (Strengthening TB and AIDS Response), and TRACK-TB were implemented in some of the same district at the same time as SPARS to strengthen medicines management. We were unable to assess the degree to which these activities might have influenced the observed results. Although the study examines changes that occurred from 2010 to 2013, studies that test methods to improve prescribing and dispensing quality at facility level remain very relevant [4]. SPARS can easily be adapted to address and monitor progress in country-specific issues; for instance, a few modifications were introduced to the SPARS AMU measures at the end of 2017 to link malaria testing results to treatment. Uganda has also now adapted a SPARS approach for building medicines management capacity within laboratory, tuberculosis, and HIV/AIDS programs and services.

## Conclusions

Building facility-level capacity in appropriate medicine use in government and PNFP health care facilities is critical for saving lives, ensuring high-quality health care services, minimizing development of antimicrobial resistance, improving patient-centered care, and optimizing use of limited resources. This study demonstrates that the multipronged SPARS approach effectively improved prescribing and dispensing practices and related infrastructure in health facilities in Uganda following 12 months of implementation. Significant improvements were observed in both behavioral and resource-dependent outcome measures. Despite overall improvement in performance, impact varied across facilities and AMU measures, so more work is needed to refine the approach. We recommend broad dissemination of the elements of the SPARS approach to strengthen appropriate medicine use in all health care facilities in Uganda and elsewhere to improve medicines management performance.

## Supplementary information

**Additional file 1.** Sections of SPARS data collection tool covering appropriate medicines use measures

**Additional file 2.** SPARS dispensing and prescribing indicators, associated measures, and definitions

**Additional file 3.** Percentage of facilities with complete measures at first visit, last visit, change from first to last visit, and complete at both first and last visits

**Additional file 4.** Characteristics of health facilities included in the analysis, overall and by level of care

**Additional file 5.** Characteristics of medicines management supervisors, overall and for the subgroup responding to a survey of their experience

## Data Availability

The data, data collection tool, interviews, analysis, and other materials are provided either in supplementary files or can be obtained from the corresponding author.

## Abbreviations

ACT: Artemisinin-based combination therapy
AMU: Appropriate medicine use
B: Behavioral change
DHO: District health officer
HC: Health center
M: Measures
MMS: Medicines management supervisors
MOH: Ministry of Health
ORS: Oral rehydration solution
PNFP: Private not-for-profit
R: Resource requiring change
RDT: Rapid diagnostic test
SPARS: Supervision, Performance Assessment, and Recognition Strategy
STGs: Standard treatment guidelines
URTI: Upper respiratory tract infection
WHO: World Health Organization
