# Supplementary material for: Article 3: 1-year impact of supervision, performance assessment, and recognition strategy (SPARS) on prescribing and dispensing quality in Ugandan health facilities
Source: J Pharm Policy Pract. 2020 Sep 1;13:48. doi: 10.1186/s40545-020-00248-w (PMC7461332; doi:10.1186/s40545-020-00248-w)
Supplement: Supplementary file 1 — Additional file 1. Sections of SPARS data collection tool covering appropriate medicines use measures [file 40545_2020_248_MOESM1_ESM.pdf]

Additional file 1: Sections of SPARS data collection tool covering appropriate medicines use measures

Dispensing quality

1. Dispensing Time

Observe and record the dispensing time for 6 patients.

Patient number	1	2	3	4	5	6	Average	Comment
Dispensing time in seconds								

Score if Average: a) <60 seconds: = 0; b) 61seconds and above we scored 1 (yes as appropriate): ______

2. Packaging Material

Observe and verify the packaging material available and in use (Yes=1/No=0)

		1/0/	Comments
A	Do you have dispensing envelopes?		
B	Do you have appropriate clean containers i.e. bottles made specifically for the purpose of dispensing liquids and bottles that are not reused?		
	Sum		

Score: the sum of a to b yes (1) divided by 2 minus NA's: ______ Percentage: ______

3. Dispensing equipment

Verify that the pharmacy(dispensary) has the following equipment in the dispensing area (Yes=1/No=0)

Equipment	1/0	Comments
a) A spatula or spoon		
b) Tablet counting tray or similar		
c) Tablets not counted by bare hands		
d) Graduated measuring cylinder		
Sum		

Sum of yes (1) of a to be divided by 5: ______ Percentage: ________

4. Services available at the dispensing area

Verify that the dispensing area/health facility has the following services (Yes=1/No=0)

Services	1/0	Comments
a) Chairs or bench to sit on in dispensing area		
b) Can privacy be achieved during dispensing?		
c) Facility to wash hands by patient in facility?		
d) Drinking water (to take tablets) by patient in dispensing area?		
Sum		

Sum of yes (1), a and b divided by 4:_______ Percentage:_______________

5. Patient care

Interview 5 patients and ask to see the medicines they have received and if possible their prescription. Select one of the medicines to check patient knowledge

	No. of medicines		Patient knowledge (Yes=1/No=0)					
Pt no.	Prescribed	Dispensed	Discrepancy (Y=1/ N=0)	Dose/ How much to take	Freq/ How often to take	Duration / how long to take	Does patient know why s/he is getting the treatment	Other information given: Adverse reactions, etc
1.								
2.								
3.								
4.								
5.								
Sum								
% (sum/5)*100			100-[(sum/5)*100] ___________	______	_______	_______	__________	Sum: ___________
Average score = Average % score of the 5 columns:								

Average % score: _______

Score if Average: a) ≥ 90% = 1; b) 75-89%=0.75; c) 50-74%=0.5; d) 30-49%= 0.25; e) <30% = 0: _____

Comments:

6. Labelling

Interview 5 patients and ask to see the medicines they have received. Select one and check for labelling (Y=1/ N=0)

Medicine no.	Medicine name*	Strength	Quantity	Date	Dose	Patient name	Facility name
1.							
2.							
3.							
4.							
5.							
Sum							
% (sum/5)*100							
Average score = Average % score of 7 columns:							___________

*Note: The medicine name appears by generic name or by brand and generic name

Score if Average score: a) ≥ 90% = 1; b) 75-89% = 0.75; c) 50-74% = 0.5; d) 30-49% = 0.25%; e) < 30% = 0: _________

Comments:

7. Non-Discrepancy between prescribed and dispensed medicines (antibiotics)

Select the last 5 entries including amoxicillin and the last 5 entries including cotrimoxazole from the prescription and dispensing log and record the amount of amoxicillin and cotrimoxazole prescribed and that dispensed, respectively.

**Drug**	**Pt. no.**	**1**	**2**	**3**	**4**	**5**	**Sum**	**Score: [1 – (sum/5)]**	**Percentage**
**Amoxicillin**	Amount prescribed								
	Amount dispensed								
	Discrepancy (Y=1/N=0)						_______	________	_________
**Cotrimoxazole**	Amount prescribed								
	Amount dispensed								
	Discrepancy (Y=1/N=0)						_____	_______	_____
Average score and percentage								_______	_______

Comments:

Score = (score of amoxicillin + cotrimoxazole)/2: _____ Percentage: ____________

Prescribing Quality

1. Correct use of prescription recording system

Record from the last 10 entries from the prescription (or OPD) and dispensing log and note whether the information below have been entered/recorded (Yes=1/No=0)

Prescription entries	Date #	OPD/IP No.	Diagnosis +	Medicines name*	Prescriber's name	Amount prescribed	Amount dispensed	Sum	% (sum/7)*100
1									
2									
3									
4									
5									
6									
7									
8									
9									
10									
Sum									________
% Average = % Sum of Last column/10									________

*+Diagnosis refers to definite diagnosis e.g. Malaria, URTI, not symptoms e.g. fever, cough; # Date should be up to date, i.e. date of the day of visit or previous day or last day the facility received patients if the previous day was a weekend; *Brand and generic name or generic name alone.*

Score if % Average: a) 100% = 1; b) 75-99% = 0.5; c) <75% = 0: _________________

Comment:

2. Rational prescribing

Randomly select 20 prescriptions from past 2 months and record information in table below (Yes=1/No=0):

**When carrying out routine supervision randomly select the 20 prescriptions from the last date of supervision*

Pt No.	No. of medicines	No. of medicines prescribed by generic name	No. of antibiotics prescribed	No. of injections prescribed	No. & name of medicines not in the Essential Medicines List of Uganda	Diagnosis recorded Diagnosis=1/ symptoms=0
1.						
2.						
3.						
4.						
5.						
6.						
7.						
8.						
9.						
10.						
11.						
12.						
13.						
14.						
15.						
16.						
17.						
18.						
19.						
20.						
Total	………..	…………….	# Patient/Drugs ………/………	# Patient/Drugs ………/……….	…………………….	# Diagnosis …………..

Average	(total/20) ……………		% Patients receiving 1 or more antibiotics ……………..	% Patients receiving 1 or more injections …………….		(D/total i.e. 20)*100 …………..
%		% of medicines prescribed by generic name ……….	% of drugs being antibiotics ……………..	% of drugs being injections ……………..	% of drugs not in the Essential Medicines List of Uganda ……………..	

Calculate:

Parameter	% or value	How to score	Score
1. Average no. of medicines prescribed per patient		≤2.5: score 0.2 else 0	
2. % of medicines prescribed by generic name		≥85%: 0.2 else 0	
3. % of patients prescribed 1 or more antibiotics		≤45%: 0.2 else 0	
4. % of patients prescribed 1 or more injections		≤45%: 0.2 else 0	
5. % Diagnosis recorded		≥85%: 0.2 else 0	
Sum of the 5:			________

Score: sum of the 5 above: _____ Percentage: ____________

Comment:

Adherence to standard treatment guidelines

Record from the prescriptions the adherence to treatment guidelines for diarrhoea, non-pneumonia respiratory tract infection (cough/cold) and uncomplicated malaria. For each condition, sample the last 10 prescriptions from the OPD book for each diagnosis. Only consider prescriptions from the last month. If there are not 10 prescriptions with that diagnosis in the last month, leave the remaining fields blank and write comment. **Only give score if more than three cases**

3. Diarrhoea (No blood)

The appropriate diagnosis is diarrhoea. Only look at prescriptions for the last month from today's date.

Disease/Drug prescribed		Cases (Yes=1, No=0)										Total/No. of cases* 100
**Diarrhoea (No blood)**		1	2	3	4	5	6	7	8	9	10	
1	ORS											
2	Antibiotics											
3	Anti-diarrhoeal											
4	Zinc											
5	Anti-spasmodic											
6	Other drugs given											Sum of A= ______
Assessment (A=1,B=0)												%A(1) = (sum/total)*100: ______

Assessment: Mark **1 for A, appropriate** (if ORS = 1, Zinc =1 or 0 and all others = 0) Else mark 0 for B;

Score: If %A (1) ≥ 50 then score =1, else 0: ____________

4. Cough/Cold

The appropriate diagnosis for this are: Non-pneumonia, ART, ARTI, common cold, flu, cough, cold, sore throat.

1	Antibiotics											
2	Antipyretic/ analgesic											
3	Cough or cold drugs											
4	Other drugs given											Sum of A= _____
**Assessment (A=1, B=0)**												%A (1) = (sum/10)*100: _____

Assessment: 1 for A, Appropriate [if antibiotics = 0 (No antibiotic given), Antipyretic/analgesic = 0 or 1 and/or Cough/Cold drugs 0 or 1; and other drugs = 0] **ELSE** not appropriate (0 for B) if Antibiotic = 1 and any other drugs given = 1.

Score: If %A (1) ≥ 50 then score =1, else 0: ____________

5. Malaria treatment (uncomplicated malaria)

Consider both malaria and clinical malaria cases. In case no test is available, score 1. ‘Rapid test or smear conducted’ with NA.

**Are Rapid Diagnostic Tests for malaria available? ☐Yes ☐No**													
**Is there a functional laboratory at the facility? ☐Yes ☐No**													
	**Testing** (1/0)	1	2	3	4	5	6	7	8	9	10	Sum	%(sum/10)*100
1	Rapid test or smear conducted											_______	___________
	Treatment (1/0)												
2	Artemether /Lumefantrine (ACT)												
3	Quinine Tab.												
4	Sulfadoxine/Pyrimethamine (SP)												
5	Antibiotics												
6	Paracetamol/Antipyretic												
7	Other drugs given												
	*Appropriate treatment (Y=1/N=0)											______	_______
**Assessment (A=1,B=0)**												A(1) = ____	%A (1) = ______

*Appropriate treatment Y=1 if ACT given or Quinine tabs (but not both), Paracetamol=0 or 1, Antibiotics=0, SP=0, other drugs=0; else N=0

Assessment: 1 for A, Appropriate: [if Mal test =1 and ACT = 1 or Mal test =1 and Quinine =1 AND Paracetamol=0 or 1, Antibiotic =0, SP=0, other drugs given=0]

Assessment: 0 for B, Not Appropriate: [if Mal test =0 and (ACT=1, or Quinine=1) or Antibiotic =1, or SP=1 or other drugs given=1]

Scoring for Malaria	%	Scoring criteria	Score
1. % Testing		If % Testing is 100%, then score 0.5, else 0	
2. % Appropriate treatment		If = 100%, then score 0.5, else 0	
3. Assessment (A=1, B=0)		If %A $\geq$ 50%, then score 1, else 0	
**Average %** (of the 3 above)			
Sum			
Overall score = (sum/2)			

Comments:
