# Supplementary material for: Article 3: 1-year impact of supervision, performance assessment, and recognition strategy (SPARS) on prescribing and dispensing quality in Ugandan health facilities
Source: J Pharm Policy Pract. 2020 Sep 1;13:48. doi: 10.1186/s40545-020-00248-w (PMC7461332; doi:10.1186/s40545-020-00248-w)
Supplement: Supplementary file 2 — Additional file 2. SPARS dispensing and prescribing indicators, associated measures, and definitions [file 40545_2020_248_MOESM2_ESM.pdf]

Additional file 2: SPARS dispensing and prescribing indicators, associated measures, and definitions

Indicator (#)	Measure number and description	Definition
**Dispensing quality**		
Dispensing time (1)	1. % of facilities with average acceptable dispensing time for 6 patients	Calculated per facility as average time with >60 sec. scored acceptable (yes): # of facilities with acceptable dispensing time/ # of facilities * 100
Packaging material (2)	2. % of facilities with dispensing envelopes<br>3. % of facilities with appropriate and clean containers	Calculated in entire sample as:<br># of facilities that meet each criterion/ # of facilities * 100
Dispensing equipment (3)	4. % of facilities with spatula or spoon<br>5. % of facilities with tablet counting tray or similar<br>6. % of facilities with tablets counted using spatula or gloves<br>7. % of facilities with graduated measuring cylinder	
Services available in dispensing area (4)	8. % of facilities with chairs/benches in dispensing area<br>9. % of facilities with privacy during dispensing<br>10. % of facilities where patients can wash hands<br>11. % of facilities with drinking water accessible to patients	
Patient care (5)	For 5 patients exiting the facility :<br>12. % of patients with all prescribed medicines dispensed<br>13. % of patients knowing dose of medicines to take<br>14. % of patients knowing how frequently to take medicines<br>15. % of patients knowing how long to take the medicines<br>16. % of patients knowing why they got the medicines	Calculated per facility as:<br># of patients who meet each criterion/ 5 * 100
Labelling (6)	For one medicine package from 5 exiting patients:<br>17. % of medicines labelled with medicines name<br>18. % of medicines labelled with strength<br>19. % of medicines labelled with quantity<br>20. % of medicines labelled with date<br>21. % of medicines labelled with dose<br>22. % of medicines labelled with patient name<br>23. % of medicines labelled with facility name	Calculated per facility as:<br># of medicines that meet each criterion/ 5 * 100
Rationing of Antibiotics (7)	Assess antibiotics dispensed for 5 patients<br>24. % with all prescribed amoxicillin dispensed<br>25. % with all prescribed cotrimoxazole dispensed	Calculated per facility as:<br># of patients who meet each criterion/ 5 * 100
**Prescribing quality**		
Prescription recording (1)	For last 10 patients recorded in dispensing/prescribing log:<br>26. % of prescriptions with date recorded<br>27. % of prescriptions with location of treatment recorded<br>28. % of prescriptions with diagnosis recorded<br>29. % of prescriptions with medicines name recorded<br>30. % of prescriptions with prescriber name recorded<br>31. % of prescriptions with amount prescribed recorded<br>32. % of prescriptions with amount dispensed recorded	Calculated per facility as:<br># of prescriptions that meet each criterion/ 5 * 100

Rational prescribing (2)	For 20 patients in dispensing/prescribing log:<br>33. Average # of medicines prescribed<br>34. % of medicines prescribed by generic name<br>35. % of patients with no antibiotic prescribed<br>36. % of patients with no injection prescribed<br>37. % of patients with diagnosis recorded<br>38. % of medicines on essential medicines list of Uganda	Calculated per facility as:<br>#33: total # of medicines prescribed/ 20<br>#34,38: # of medicines meeting each criterion/ total # of medicines * 100<br>#35-37: # of patients meeting each criterion/ 20 * 100
STG for diarrhea (3)	For 10 patients with watery (non-bloody) diarrhoea in dispensing/prescribing log:<br>39. % of patients prescribed oral rehydration salt (ORS)<br>40. % of patients prescribed ORS and no antibiotics<br>41. % of patients prescribed ORS and no anti-diarrhoeal<br>42. % of patients prescribed ORS and zinc<br>43. % of patients prescribed ORS and no antispasmodic<br>44. % of patients with appropriate prescription for diarrhoea*	Calculated per facility as:<br># of patients who meet each criterion/ 10 * 100
STG for cough/ cold (4)	For 10 patients diagnosed with cough/cold (mild respiratory tract infection, common cold, flu, cough, cold, sore throat):<br>45. % of patients prescribed no antibiotics<br>46. % of patients prescribed antipyretic/analgesic<br>47. % of patients prescribed cough or cold medicines<br>48. % of patients with appropriate prescription for cough/cold**	Calculated per facility as:<br># of patients who meet each criterion/ 10 * 100
STG for malaria (5)	Infrastructure for malaria treatment:<br>49. % of facilities with Rapid Diagnostic Tests (RDT) available<br>50. % of facilities with functional lab for malaria testing	Calculated in sample as:<br># of facilities that meet each criterion/ # of facilities * 100
	For 10 patients diagnosed with fever or malaria:<br>51. % of patients with malaria RDT or smear conducted<br>52. % of patients receiving artemisinin combination therapy<br>53. % of patients receiving quinine tablet<br>54. % of patients receiving no sulfadoxine/ pyrimethamine (SP)<br>55. % of patients receiving no antibiotics<br>56. % of patients receiving paracetamol/antipyretics<br>57. % patients with appropriate treatment for malaria***	Calculated per facility as:<br># of patients who meet each criterion/ 10 * 100

*Appropriate prescribing for diarrhoea is prescription of oral rehydration salt with and without zinc.

** Appropriate prescription for cough/cold (mild or moderate upper respiratory tract infection) is that no antibiotics are given, Antipyretic/analgesic or cough /cold medicines might be given.

*** Appropriate treatment for malaria is prescription of artemisinin combination therapy or Quinine tablets but not both. Paracetamol might or might not be given but no antibiotics and no sulfadoxine/pyrimethamine.
