# Supplementary material for: Article 3: 1-year impact of supervision, performance assessment, and recognition strategy (SPARS) on prescribing and dispensing quality in Ugandan health facilities
Source: J Pharm Policy Pract. 2020 Sep 1;13:48. doi: 10.1186/s40545-020-00248-w (PMC7461332; doi:10.1186/s40545-020-00248-w)
Supplement: Supplementary file 3 — Additional file 3. Percentage of facilities with complete measures at first visit, last visit, change from first to last visit, and complete at both first and last visits [file 40545_2020_248_MOESM3_ESM.pdf]

Additional file 3: Percentage of facilities with complete measures at first visit, last visit, change from first to last visit, and complete at both first and last visits

AMU measure	Type *	Item completeness			
		First visit (%)	Last visit (%)	Change (%)	Both visits (%)
**Dispensing quality**					
1. % acceptable dispensing time	B	98%	99%	1%	97%
2. % of facilities with dispensing envelopes	R	100%	100%	0%	100%
3. % of facilities with appropriate and clean containers	R	100%	100%	0	100%
4. % of facilities with spatula or spoon	R	99.9%	100.0%	0%	100%
5. % of facilities with tablet counting tray or similar	R	99.9%	100.0%	0%	100%
6. % of facilities with tablets counted using spatula or gloves	R	99.9%	100.0%	0%	100%
7. % of facilities with graduated measuring cylinder	R	99.9%	100.0%	0%	100%
8. % of facilities with chairs/benches in dispensing area	R	99.8%	99.8%	0%	100%
9. % of facilities with privacy during dispensing	R	99.8%	99.9%	0%	100%
10. % of facilities where patients can wash hands	R	99.8%	99.9%	0%	100%
11. % of facilities with drinking water accessible to patients	R	99.8%	99.9%	0%	100%
12. % of patients with all prescribed medicines dispensed	B	96.6%	98.0%	1%	95%
13. % of patients knowing dose of medicines to take	B	96.2%	98.0%	2%	95%
14. % of patients knowing how frequently to take medicines	B	96.2%	98.0%	2%	95%
15. % of patients knowing how long to take the medicines	B	96.2%	98.0%	2%	95%
16. % of patients knowing why they got the medicines	B	96.2%	98.0%	2%	95%
17. % of medicines labelled with medicines name	B	96.2%	98.0%	2%	95%
18. % of medicines labelled with strength	B	96.8%	97.8%	1%	95%
19. % of medicines labelled with quantity	B	96.8%	97.8%	1%	95%
20. % of medicines labelled with date	B	96.8%	97.8%	1%	95%
21. % of medicines labelled with dose	B	96.8%	97.8%	1%	95%
22. % of medicines labelled with patient name	B	96.8%	97.8%	1%	95%
23. % of medicines labelled with facility name	B	96.8%	97.7%	1%	95%
24. % with all prescribed amoxicillin dispensed	R	50.6%	76.4%	26%	42%
25. % with all prescribed cotrimoxazole dispensed	R	55.2%	84.7%	29%	50%
**Prescribing quality**					
26. % of prescriptions with date recorded	B	95.4%	97.8%	2%	94%
27. % of prescriptions with location of treatment recorded	B	95.4%	97.8%	2%	94%
28. % of prescriptions with diagnosis recorded	B	95.4%	97.8%	2%	94%
29. % of prescriptions with medicines name recorded	B	95.4%	97.8%	2%	94%
30. % of prescriptions with prescriber name recorded	B	95.3%	97.7%	2%	94%
31. % of prescriptions with amount prescribed recorded	B	95.4%	97.7%	2%	94%
32. % of prescriptions with amount dispensed recorded	B	95.1%	97.4%	2%	93%
33. Average # of medicines prescribed	B	96.6%	98.0%	1%	94%
34. % of medicines prescribed by generic name	B	96.6%	98.0%	1%	95%
35. % of patients with no antibiotic prescribed	B	99.0%	99.5%	0%	99%
36. % of patients with no injection prescribed	B	99.0%	99.5%	0%	99%
37. % of patients with diagnosis recorded	B	98.7%	98.9%	0%	98%
38. % of medicines on essential medicines list of Uganda	B	98.4%	98.9%	1%	97%
39. % of patients prescribed oral rehydration salt (ORS)	B	86.4%	81.8%	-5%	74%
40. % of patients prescribed ORS and no antibiotics	B	86.4%	80.9%	-5%	73%
41. % of patients prescribed ORS and no anti-diarrhoeal	B	86.3%	81.1%	-5%	73%
42. % of patients prescribed ORS and zinc	B	73.1%	80.1%	7%	62%

AMU measure	Type *	Item completeness			
		First visit (%)	Last visit (%)	Change (%)	Both visits (%)
43. % of patients prescribed ORS and no antispasmodic	B	86.3%	80.3%	-6%	72%
44. % of patients with appropriate prescription for diarrhoea	B	86.3%	81.8%	-5%	74%
45. % of patients prescribed no antibiotics	B	94.7%	94.5%	0%	90%
46. % of patients prescribed antipyretic/analgesic	B	94.7%	95.1%	0%	91%
47. % of patients prescribed cough or cold medicines	B	94.7%	94.7%	0%	91%
48. % of patients with appropriate prescription for cough/cold	B	83.0%	84.3%	1%	72%
49. % of facilities with Rapid Diagnostic Tests (RDT) available	R	99.2%	99.2%	0%	99%
50. % of facilities with functional lab for malaria testing	R	86.9%	66.1%	-21%	63%
51. % of patients with malaria RDT or smear conducted	B	81.8%	84.2%	2%	78%
52. % of patients receiving artemisinin combination therapy	B	96.6%	98.4%	2%	95%
53. % of patients receiving quinine tablet	B	96.3%	97.2%	1%	94%
54. % of patients receiving no pyrimethamine/sulfadoxine SP	B	96.3%	97.0%	1%	94%
55. % of patients receiving no antibiotics	B	96.3%	97.3%	1%	94%
56. % of patients receiving paracetamol/antipyretics	B	96.3%	98.4%	2%	95%
57. % patients with appropriate treatment for malaria	B	96.6%	98.5%	2%	95%
