# Supplementary material for: Article 3: 1-year impact of supervision, performance assessment, and recognition strategy (SPARS) on prescribing and dispensing quality in Ugandan health facilities
Source: J Pharm Policy Pract. 2020 Sep 1;13:48. doi: 10.1186/s40545-020-00248-w (PMC7461332; doi:10.1186/s40545-020-00248-w)
Supplement: Supplementary file 4 — Additional file 4. Characteristics of health facilities included in the analysis, overall and by level of care [file 40545_2020_248_MOESM4_ESM.pdf]

Additional file 4: Characteristics of health facilities included in the analysis, overall and by level of care

Study facilities	Total		HC2		HC3		HC4/hospital		χ^2
	No.	%	No.	%	No.	%	No.	%	p-value
	1222	100	681	56	416	34	125	10	
Region									
Central	250	21	133	20	92	22	25	20	0.343
Western	421	35	224	33	145	35	52	42	
Eastern	379	31	226	33	118	28	35	28	
Northern	172	14	98	14	61	15	13	10	
Ownership									
Government	1039	85	596	88	349	84	94	75	0.002
PNFP	183	15	85	13	67	16	31	25	
Year of initial visit									
2011	753	62	368	54	289	70	96	77	<0.001
2012	406	33	263	39	117	28	26	21	
2013	63	5	50	7	10	2	3	2	
Number of visits in first year									
2	328	27	184	27	115	28	29	23	
3	334	27	176	26	115	28	43	34	
4	323	26	180	26	108	26	35	28	
5	201	16	122	18	62	15	17	14	
6	35	3	19	3	15	4	1	1	
7	1	0	0	0	1	0	0	0	
Health workers supervised at initial visit									
One	280	23	223	33	45	11	12	10	<0.001
More than one	942	77	458	67	371	89	113	90	
MMS supervising during initial visit									
One	957	78	603	89	292	70	62	50	<0.001
More than one	265	22	78	12	124	30	63	50	
Designated MMS supervised initial visit*									
No	394	32	208	31	150	36	36	29	0.118
Yes	828	68	473	69	266	64	89	71	

*Designated MMS is the MMS assigned to a facility who was responsible for a majority of visits
