# Supplementary material for: Article 3: 1-year impact of supervision, performance assessment, and recognition strategy (SPARS) on prescribing and dispensing quality in Ugandan health facilities
Source: J Pharm Policy Pract. 2020 Sep 1;13:48. doi: 10.1186/s40545-020-00248-w (PMC7461332; doi:10.1186/s40545-020-00248-w)
Supplement: Supplementary file 5 — Additional file 5. Characteristics of medicines management supervisors, overall and for the subgroup responding to a survey of their experience [file 40545_2020_248_MOESM5_ESM.pdf]

Additional file 5: Characteristics of medicines management supervisors, overall and for the subgroup responding to a survey of their experience

Characteristics	No.	%
MMS study total	148	100
Gender		
Male	124	84
Female	24	16
Level		
District MMS	53	36
Sub district MMS	95	64
Regions		
Central	31	21.0
Western	56	37.8
Eastern	41	27.7
Northern	20	13.5
Facilities supervised		
1-10	81	54.7
11-15	47	31.7
16+	20	13.6
Professional training		
Clinical officer	87	59
Pharmacist/dispenser	15	10
Nurse	36	24
Supplies officer	10	7

Characteristics	No.	%
MMS completing 2013 survey	111	75
Age group		
26-35	37	34
36-45	46	42
46+	26	24
Highest level of education		
Secondary/diploma/other	92	83
Bachelors/Master's degree	19	17
Number of years of work experience		
0-9	45	40
10+	66	60
Frequency of meetings with DHO		
Monthly/weekly	60	54
Quarterly/semi-annually	24	22
Irregularly/other	27	24
Received feedback from DHO about MMS report		
No	16	15
Yes	92	85
Sufficient time during visits to provide adequate supportive supervision		
No	38	35
Yes	71	65
Health workers respond well to supervision		
Some of them	40	37
Most/all of them	68	63
